# Visual Rating Scales of White Matter Hyperintensities and Atrophy: Comparison of Computed Tomography and Magnetic Resonance Imaging

**DOI:** 10.1016/j.jstrokecerebrovasdis.2018.02.028

**Published:** 2018-07

**Authors:** Karen J. Ferguson, Vera Cvoro, Alasdair M.J. MacLullich, Susan D. Shenkin, Peter A.G. Sandercock, Eleni Sakka, Joanna M. Wardlaw

**Affiliations:** * Edinburgh Delirium Research Group, Geriatric Medicine, University of Edinburgh, Edinburgh, UK; † Centre for Cognitive Ageing and Cognitive Epidemiology, University of Edinburgh, Edinburgh, UK; ‡ Victoria Hospital, NHS Fife, Kirkcaldy, UK; § Centre for Clinical Brain Sciences, University of Edinburgh, Edinburgh, UK; ‖ Brain Research Imaging Centre, University of Edinburgh, Edinburgh, UK; ¶ UK Dementia Research Institute at the University of Edinburgh, Edinburgh, UK

**Keywords:** Computed tomography, magnetic resonance imaging, cerebral atrophy, white matter lesions, validity

## Abstract

**Goal:**

Magnetic resonance imaging (MRI) is the preferred modality for research on structural age-related brain changes. However, computed tomography (CT) is widely available and has practical and cost advantages over MRI for large-scale brain imaging research studies in acutely unwell patients. However, the relationships between MRI and CT measures of white matter hyperintensities (WMH) and atrophy are unclear. We examined the relationships between visual ratings of WMH, atrophy, and old infarcts in patients who had both CT and MRI scans.

**Materials and Methods:**

Patients who had both CT and MRI scans in the International Stroke Trial-3 were studied. In both modalities, 2 raters independently completed standardized visual rating scales for WMH, and for central and superficial atrophy using a 5-point scale. In addition, 1 rater recorded old infarcts according to size and location.

**Findings:**

Seventy patients with a mean age of 69 years were studied. There were moderate to substantial intrarater CT–MRI agreements for periventricular components of WMH scales (weighted Κappa = .55-.75). Agreements for basal ganglia ratings were lower (weighted Κappa = .18-.44), partly because of the misclassification of prominent perivascular spaces. Atrophy scales showed moderate to substantial CT–MRI agreements (weighted Κappa = .44-.70). MRI was more sensitive in the detection of smaller infarcts and cavitated lesions.

**Conclusions:**

Standardized visual rating scales of white matter lesions and atrophy mostly show substantial agreement between CT and MRI. Clinical CT scans have a strong potential for wider exploitation in research studies, particularly in acutely unwell populations.

## Introduction

**Figure 1:**
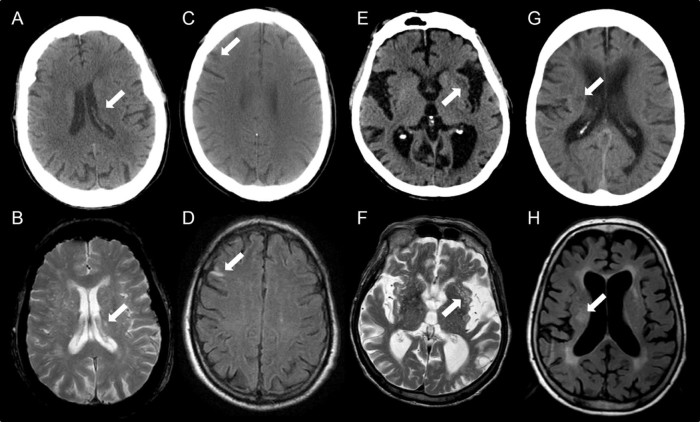
Examples of misclassification of old infarcts between CT and MRI. A small lacunar infarct was just visible on CT (A) but below the size threshold for recording. The same lesion appeared larger on T_2_w MRI (B). A small cortical lesion missed on CT (C) was obvious on FLAIR MRI due to tissue signal changes (D). Low attenuation in the external capsule was recorded as an old infarct on CT (E) but was found to represent a cluster of EPVS on T_2_w MRI (F). A focal area of low attenuation observed on CT was identified as a noncavitated lacunar lesion (G); this was considered to be a WMH on FLAIR MRI (H). All assessments of old infarct agreement was based on data from 1 rater.

Computed tomography (CT) is very commonly performed in older patients admitted to hospital with acute conditions such as stroke, reduced level of consciousness, and delirium. Magnetic resonance imaging (MRI) provides advantages over CT with respect to resolution, gray–white matter differentiation, and range of modalities, but it is clinically used less frequently, especially in acutely unwell populations. This is because CT is comparatively better tolerated, more rapid, less expensive, and more available than MRI. With the advent of the systematic electronic collection of clinical data (e.g., cognitive status) and the availability of data linkage, there is an enormous unfulfilled potential for exploitation of the large numbers of routinely performed CT scans in relation to acute and long-term clinical outcomes. Recent studies provide evidence of the value of cross-sectional and longitudinal CT scanning in predicting outcomes and understanding the neural substrates. For example, in stroke patients, cerebral atrophy on routine CT scans was associated with a higher risk of institutionalization at 3 years,^1^ while pre-existing signs on CT predicted reduced independence at 6 months and increased symptomatic hemorrhage post–ischemic stroke^2^ (Figure 1).

CT measures of atrophy and white matter lesions have been evaluated against MRI reference standards. For example, 1 study found very high correlations between CT and MRI measurements of perihemorrhagic edema volumes after intracerebral hemorrhage (Pearson correlation coefficient R^2^ = .96, slope of 1.05, *P* < .01), although cerebral atrophy was not assessed.^3^ High intraobserver agreements were found for ratings of global cerebral atrophy (Kappa = .83), medial temporal lobe atrophy (Kappas = .88 [left], .86 [right], and Fazekas' subcortical WMH .79) between MRI and CT in 31 memory clinic outpatients.^4^ Other studies report comparable findings.^5^, ^6^, ^7^ However, the evidence base remains small, and there remains uncertainty over the relationships between CT and MRI measures of white matter hyperintensities (WMH) and atrophy. Therefore, here we compared qualitative assessment of WMH, atrophy, and old infarcts in a group of patients in the third International Stroke Trial who had undergone both CT and MRI scanning within the acute phase of their illness.

## Materials and Methods

### Participants

We used anonymized CT and MRI scans from the International Stroke Trial-3 (IST-3), an international multicenter, randomized controlled trial. The IST-3 study was approved by the Scotland Research Ethics Committee and by ethics committees and other regulatory bodies of all participating countries, hospitals, and institutions. All patients, or a legal proxy if the patient lacked capacity, provided written informed consent.

### Imaging

We used 70 paired CT and MRI scans performed clinically and as part of research, with a short delay (hours or a few days) between CT and MRI (CT always first). Anonymized clinical spiral or multislice brain CT and MRI including T_2_, FLAIR, and DTI sequences were stored digitally and analyzed on a Picture Archiving and Communication System. Raters were blind to MRI scans at the time of CT analysis and vice versa. All CT scans were analyzed first followed by all MRI scans several weeks later. For the intrarater reliability calculations, 1 rater (K.F.) repeated the CT analysis months after the initial scoring and was blind to the original results.

### Analyses

Visual ratings by 2 raters (K.F. and V.C.) included deep and superficial atrophy, which were scored separately according to a previously validated 5-point scheme.^8^ In addition, WMHs were rated according to the Age-Related White Matter Change (ARWMC) scale (frontal, parieto-occipital, and basal ganglia all rated on 4-point scales; the temporal and infratentorial aspects of this scale were omitted), van Swieten's scale (anterior and posterior periventricular lesions rated separately on 3-point scales), and Fazekas' scale.^5^, ^9^, ^10^ Although Fazekas' scale was devised in MRI, we applied it in the same way to CT: this scale comprises two 4-point scales for assessing periventricular lesions and deep WMHs (0, absence; 1, cap of pencil-thin PVH or punctate focal deep WMH; 2, smooth halo PVH or early confluence of focal deep WMH; 3, irregular PVH extending into the deep white matter or large confluent areas of deep WMH). Old infarct recording was performed by 1 experienced neuroimaging research scientist (K.F.) according to a scheme devised for coding the location and extent of old and acute strokes.^2^, ^11^ Criteria for lacunar infarcts were (1)  ≥3, ≤20 mm (maximal dimension) and (2) typical location, for example in subcortical gray matter, internal or external capsule, or deep white matter as well as cerebellum and brain stem. The volume of presumed lacunar infarcts was calculated by multiplying the maximal dimensions in 2 perpendicular planes on an axial slice, the number of slices in which the lesion occurred and the slice thickness.

### Statistical Analysis

Weighted Kappa values with linear weighting were used (IBM SPSS Statistics for Windows, Version 19.0; IBM Corp., Armonk, NY), and agreement was interpreted according to Landis and Koch:  <0 = less than chance; .01-.20 = slight; .21-.40 = fair; .41-.60 = moderate; .61-.80 = substantial; .81-.99 = almost perfect.^12^ McNemar's test of marginal homogeneity (MH by Uebersax, http://www.john-uebersax.com/stat/mh.htm) were also applied to examine the symmetry between CT and MRI scores and for inter-rater reliability within CT.

## Results Sample

The median age of the patients was 76.66 years (interquartile range = 27.48), and 43 were men (61%).

## Visual Rating Scales

Weighted Kappa statistics for agreement between CT and MRI, and inter-rater and intrarater reliabilities are shown in Table 1.

**Table 1:** Weighted Kappa statistics for agreement between CT and MRI, and inter-rater and intrarater reliabilities

	Rater 1 CT versus MRI (95% CI)	Rater 2 CT versus MRI (95% CI)	Rater 1 versus Rater 2 inter-rater within CT (95% CI)	Rater1 intrarater within CT (95% CI)
ARWMC frontal	.58 (.45-.72)	.70 (.60-.81)	.74 (.63-.84)	.73 (.61-.85)
ARWMC parieto-occipital	.61 (.49-.74)	.65 (.53-.77)	.75 (.64-.86)	.73 (.62-.83)
ARWMC basal ganglia	.44 (.27-.60)	.18 (.02-.34)	.28 (.13-.42)	.55 (.40-.70)
van Swieten anterior	.67 (.52-.81)	.60 (.44-.75)	.65 (.52-.79)	.78 (.66-.90)
van Swieten posterior	.66 (.53-.79)	.62 (.47-.77)	.63 (.51-.75)	.69 (.57-.81)
Fazekas periventricular	.68 (.55-.80)	.55 (.42-.68)	.69 (.59-.80)	.75 (.64-.85)
Fazekas deep white matter	.57 (.43-.70)	.70 (.58-.81)	.50 (.35-.64)	.67 (.54-.79)
Superficial atrophy	.61 (.49-.73)	.43 (.25-.61)	.45 (.32-.58)	.65 (.53-.77)
Deep atrophy	.70 (.59-.81)	.53 (.40-.66)	.47 (.34-.60)	.65 (.54-.77)

Abbreviations: ARWMC, Age-Related White Matter Change; CT, computed tomography; MRI, magnetic resonance imaging.

Kappas (95% CI) with linear weighting showing agreement for both raters between CT and MRI, inter-rater agreement within CT, and intrarater agreement for WMH and atrophy scores. Intra-rater reliability for CT scoring was performed by 1 rater (Rater 1).

### CT Versus MRI

For both raters, weighted Kappas indicated moderate to substantial agreement between most WMH and most atrophy scores between CT and MRI (Κ = .43-.70 for Raters 1 and 2). The exception was the ARWMC basal ganglia scores for Rater 2, which showed lower agreement between CT versus MRI with consistently higher MRI scoring compared with CT. A lower Kappa for this scale (Κ = .44) by Rater 1 compared with the other WMH scales also reflected higher MRI scores. McNemar's tests were nonsignificant for CT versus MRI scoring by Rater 1. McNemar's tests were significant for most scales for Rater 2 (ARWMC frontal: χ^2^ = 4.55; *P* = .033; ARWMH parieto-occipital: χ^2^ = 5.54; *P* = .018; ARWMH basal ganglia: χ^2^ = 13.33; *P* = .0003; Van Swieten anterior: χ^2^ = 8.00; *P* = .0047; Van Swieten posterior: χ^2^ = 8.90; *P* = .0029; Fazekas' periventricular: χ^2^ = 8.00; *P* = .0047; Fazekas' deep white matter: χ^2^ = 5.26; *P* = .022) consistent with higher scoring on MRI by this rater compared with CT.

### CT Versus CT (Inter-Rater)

Inter-rater agreements within CT were substantial for periventricular aspects of all the scales used (Κ = .63-.75). Moderate agreement was achieved in Fazekas' deep WMH scores and for both atrophy scales (Κ = .50, .45, and .47). The ARWMC basal ganglia scores showed fair agreement (Κ = .28) attributed to higher scoring within CT by Rater 1 compared with Rater2. McNemar's tests were significant for some scales (ARWMH basal ganglia: χ^2^ = 26.13, *P* < .001; Van Swieten anterior: χ^2^ = 17.00, *P* < .001; Van Swieten posterior: χ^2^ = 12.56, *P* = .0014; superficial atrophy: χ^2^ = 18.67, *P* < .001), reflecting higher rating by Rater 1 compared with Rater 2.

### CT Versus CT (Intra-Rater)

Intrarater agreements were mostly in the range of Kappa = .65-.78, indicating substantial agreement except for the ARWMC basal ganglia score, which showed moderate agreement (Kappa = .55). Higher scores were given generally when rated for the first time, resulting in several significant McNemar's tests (ARWMC basal ganglia: χ^2^ = 4.17, *P* = .041; posterior van Swieten: χ^2^ = 9.80, *P* = .0017; periventricular Fazekas: χ^2^ = 5.00, *P* = .025; and deep Fazekas' scores: χ^2^ = 6.00, *P* = .0014), possibly reflecting a training effect. A better intrarater basal ganglia agreement compared with inter-rater comparisons suggests a difference in the application of the scale by the 2 raters.

## Old Infarcts

One rater determined that 47 patients exhibited old infarcts, with 130 lesions in total coded on MRI, CT, or both modalities. Of these lesions, 27 were cortical and 103 lacunar (54 cavitated). Table 2 shows the frequency of agreement for cortical infarcts and cavitated and noncavitated lacunes. Cortical lesions showed the highest rate of “perfect agreement” in both MRI and CT (48%) followed by cavitated lacunar lesions (33%). Only 10% of noncavitated lacunar lesions were correctly identified on both MRI and CT. The most frequent reason for failure to identify lesions was size, with 26% of cortical lesions, 33% of noncavitated lacunar lesions, and 35% of cavitated lacunar lesions identified on MRI but not visible or (in the case of lacunes) below the size threshold (≤3 mm) for inclusion on CT. Analysis of volume data for all lacunar lesions indicated that lesions observed on both modalities had a mean volume of 314.38 mm^3^ when measured on MRI, but the mean volume of the same lesions measured on CT was only 239.62 mm^3^, consistent with the lower sensitivity of CT to subtle tissue changes.

**Table 2:** Agreement between CT and MRI coding for cortical, noncavitated lacunar, and cavitated lacunar lesions

	Cortical	Noncavitated lacunar	Cavitated lacunar
Perfect agreement	48	10	33
Identified as an infarct on both modalities but coded differently (territory or size)	11	2	9
Visible on both modalities but identified as an infarct on 1 only (e.g., confusion with WMH)	4	41	17
Not coded on 1 modality (below size threshold or not visible)	26	41	39
Confusion between old/acute	11	4	2
Missed (identified on review)	0	2	0

Abbreviations: CT, computed tomography; MRI, magnetic resonance imaging; WMH, white matter hyperintensity.

The frequency (expressed as percentages) of coding agreement or misclassification for cortical, noncavitated lacunar, and cavitated lacunar lesions.

The mean volume of lesions identified on MRI but undetected on CT was very small at 107.23 mm^3^. Some infarcts were observed in both modalities but were coded differently due to a perceived different location or extent, with lesions appearing larger on MRI (11% cortical, 2% noncavitated lacunar, 9% cavitated lacunar). Other lesions were observed on both modalities but were only classed as infarcts on 1. This occurred most frequently where lacunes observed on CT were not recorded as WMH on MRI, accounting for 41% of presumed noncavitated and 17% of cavitated lesions. In other situations, basal ganglia “infarcts” were recorded on CT but on MRI were found to be clusters of enlarged perivascular spaces (PVS). There was some confusion between old lesions and acute lesions, particularly cortical infarcts of which 11% were misclassified on CT, with DWI MRI allowing better discrimination.

## Discussion

The main findings of this study are that standardized visual rating scales of white matter lesions and atrophy mostly show good agreement between CT and MRI. Additionally, larger and cavitated old infarcts seen on MRI could be reliably identified on CT. Only 1 measure (ARWMC scale basal ganglia rating) was performing at less-than-moderate agreement for intrarater analysis on comparing the different modalities and also for inter-rater results on CT. This is the largest study examining visual ratings of atrophy, and is of comparable size to a prior study of visual ratings in CT and MRI^5^ and also adds new analyses of CT versus MRI in the detection and classification of old infarcts. The present findings support the use of clinical CT scans as a research tool assessing how white matter lesions and brain atrophy contribute to risk and outcomes. The use of clinical CT has particular value in studies of large numbers of acutely unwell patients in which MRI studies would not be practical.

Some scales showed better inter-rater agreement between CT and MRI than others. In particular, subscales for rating periventricular lesions appeared to be more reliable. This is perhaps because periventricular lesions are easier to visualize and the areas of low attenuation surrounding the ventricles are less ambiguous than lesions in the deep white matter and basal ganglia. In contrast, there was poorer agreement in scoring where deep gray tissue was rated separately, such as the ARWMC basal ganglia scores. In these areas, PVS are common, are concomitant with other features of small vessel disease,^13^ and are easily visualized on T_2_–weighted MRI but are difficult to resolve on CT. When PVS are prevalent, “normal” WM appears less attenuated on CT and clusters of PVS may resemble larger lesions, making interpretation in deeper areas harder. Tissue signal changes surrounding lesions may make lesions on MRI appear confluent, whereas they seem discrete on CT. In addition, differences in the interpretation of the scale, with ambiguous definitions for some grades such as “beginning of confluence of foci,” may have reduced consistency between raters. A recent study^14^ in which ambiguity was reduced by providing more specific operational definitions for each score in the ARWMC scale resulted in improved reliability compared with the performance of the original scale (for example, for inter-rater reliability on CT Kappa = .87 for the operational scale compared with Kappa = .57 for the original scale).

Higher sensitivity to WMH on FLAIR reduces agreement between CT and MRI. Consistent with this, previous studies demonstrated increased sensitivity for WMH detection by MRI.^6^, ^7^ In the present study, Rater 2 consistently rated MRI higher as confirmed by significant McNemar's tests. Interestingly, inter-rater agreement for within-CT scoring tended to be higher than CT versus MRI, suggesting that differences between raters contributed to discrepancies less than variations between the modalities.

Kappa values tended to be lower for atrophy ratings than most WMH scoring. The 5-point scale used for atrophy measures here results in a greater range in scores than in the other scales, possibly affecting Kappa values. One published study^4^ achieved better agreement in atrophy rating, although this study used a 4-point scale and assessed atrophy globally rather than distinguishing between deep and superficial aspects.

Larger cortical and cavitated lacunar lesions were reliably seen on both CT and MRI scans. However, smaller lesions tended to be missed on CT possibly due to the low threshold of inclusion for lacunar lesions (≥3 mm) and lower sensitivity to small cortical lesions. Lesions appear larger on MRI because it is more sensitive to subtle changes in the surrounding tissue. This was apparent when comparing the calculated volumes of the same lesions on both modalities (239.62 mm^3^ in CT versus 314.38 mm^3^ in MRI). Some smaller cortical lesions were visible on MRI due to signal intensity changes, but with little discernible structural change, they were difficult to detect on CT. Identification of noncavitated lesions on CT was problematic with a high degree of overlap with WMH. This replicates findings of a survey of researchers of small vessel disease in which cavitated lesions (lacunes) were more likely to be identified as lacunar stroke lesions than noncavitated lesions (63% versus 7.8%, respectively) and noncavitated lesions were only classified as lacunar strokes if they were large and if WMHs were absent.^15^

Simplification of coding (fewer categories, collapsing territories) may also improve reliability for nonexperts or routine clinical applications where location is of less relevance than simply identifying that a lesion exists and its type. In the method used in this study, there are currently different codes for basal ganglia, thalamic, centrum semiovale, and internal border zone lacunar infarcts derived from a research classification and relevant to future studies of delirium or dementia and lesion location. However, reduced contrast among deep structures on CT and differences in scan orientation can make correct coding difficult. Additionally, determining the size of cortical infarcts can be subjective and lead to errors, particularly in the case of cortical infarcts that appear larger on MRI due to changes in surrounding tissue. Also on CT, while many acute and old lesions can be easily differentiated, less expert reviewers may find it more difficult to differentiate some old from some recent lesions, whereas on MRI diffusion imaging makes it very obvious which is the acute lesion. The error would not be relevant in a nonstroke population.

There were some limitations in this study. Kappa statistics were used to evaluate the magnitude of agreement. A limitation of Kappa is that it is susceptible to the prevalence of observations, number of categories, and sample size. The tests of marginal homogeneity indicated symmetry and bias but did not give an indication of the magnitude of agreement. The scans analyzed in this study were performed in acute stroke patients, and there was mild movement artifact in some scans (although very poor scans were excluded), yet despite this it was still possible to analyze all the images, but scan quality may have accounted for some discrepancy in grading between CT and MRI scans. On the other hand, this is typical of most delirious or long-term cognitively impaired patients at hospital admission.

In conclusion, the present findings facilitate greater exploitation of clinical CT in studying atrophy and white matter lesions in acute illness or in patients with contraindications to MRI. Multiple scientific questions could be tested, including (1) global and regional atrophy and WMHs as predictors of clinical outcome; (2) associations between old infarcts and clinical outcome; (3) longitudinal change (with second scans); and (4) CT measures of brain pathology as independent determinants of longer-term outcomes (e.g., institutionalization). Therefore, CT may be an important tool for understanding the neural substrates of conditions that affect older people and are difficult to research using MRI. The identification of more robust visual rating methods could potentially improve consistency and reliability in the reporting of clinical scans.
